# Tanshinone IIA Alleviates CCL2-Induced Leaning memory and Cognition Impairment in Rats: A Potential Therapeutic Approach for HIV-Associated Neurocognitive Disorder

**DOI:** 10.1155/2020/2702175

**Published:** 2020-02-22

**Authors:** Yuan-jun Liao, Jian-min Chen, Jiang-yi Long, Yi-jun Zhou, Bing-yu Liang, Yan Zhou

**Affiliations:** ^1^Department of Pharmacology, Guangxi Medical University, Nanning, Guangxi 530021, China; ^2^Guangxi Key Laboratory of AIDS Prevention and Treatment, Guangxi Medical University, Nanning, Guangxi 530021, China

## Abstract

Chemokine CC motif ligand 2 (CCL2) is one of the most recognized proinflammatory chemokines, and the expression of CCL2 in the cerebrospinal fluid of patients infected with HIV-1 is significantly higher than that of healthy people. As such, it is seen as an important cause of HIV-associated neurocognitive disorder (HAND). Our previous investigation has confirmed the pathological role of CCL2 in mediating brain damage leading to cognitive dysfunction. Currently, however, research on therapeutic drugs for the central nervous system targeting CCL2 is very limited. Our present study used brain stereotactic technology to induce cognitive impairment in rats by injecting CCL2 (5 ng) into the bilateral hippocampus. To investigate the protective effect and mechanism of Tanshinone IIA (25, 50, 75 mg/kg/d) on CCL2-induced learning memory and cognitive impairment in rats, we performed the Morris water maze (MWM) and novel object recognition tests (NORT) on the rats. The results showed that Tanshinone IIA significantly alleviated CCL2-induced learning memory and cognitive dysfunction. Further studies on the hippocampal tissue of the rats revealed that Tanshinone IIA treatment significantly increased the activity of SOD and GSH-Px while the level of MDA decreased compared to the model group. Additionally, the relative expression of apoptosis-associated genes caspase-3, caspase-8, and caspase-9 and inflammation-associated genes IL-1*β* and IL-6 in Tanshinone IIA-treated rats was lower than that in model rats. Finally, we confirmed hippocampal neuron loss and apoptosis by Nissl staining and TdT-mediated dUTP Nick end labeling (TUNEL). Taken together, these data imply that Tanshinone IIA can ameliorate CCL2-induced learning memory and cognitive impairment by impacting oxidative stress, inflammation, and apoptosis. Tanshinone IIA may be a potential therapeutic agent for the treatment of HAND.

## 1. Introduction

The consequence of progressive human immunodeficiency virus type 1 (HIV-1) infection often includes cognitive, behavioral, and neurological impairments, termed collectively as HIV-associated neurocognitive disorder (HAND) [1]. Three different categories of HAND have so far been described: asymptomatic neurocognitive impairment (ANI), HIV-1-associated mild neurocognitive disorder (MND), and HIV-1-associated dementia (HAD), which is the most severe manifestation of HAND [2]. HIV-1 infiltrates the central nervous system (CNS) in the early stage after systemic infection [3]. The incidence of HAD decreased dramatically after the advent of combination antiretroviral therapy (cART), from 20% in adults and 50% in children to less than 5% of all infected subjects [4]. Although the introduction of cART has significantly decreased the incidence of HAD, the prevalence of less severe forms of HAND has been on the rise even in the cART era [5]. In addition, the growth of antiretroviral drug resistance and changes in the AIDS dementia complex in the era of highly active antiretroviral therapy (HAART), as well as newly extended lifespans in patients with AIDS, may increase HAND prevalence [6]. This indicates that HAND will continue to be a significant complication of advanced HIV-1 infection. As HAART cannot provide complete protection from HIV-1-associated neuronal damage, there is no efficacious treatment for HAND at present. Studies have shown that HIV-1-infected cells produce soluble immune and inflammatory factors with neurotoxic potential, leading to neural injury and HAND pathogenesis. Among the potential neurotoxic factors is chemokine CC motif ligand 2 (CCL2), also known as monocyte chemoattractant protein 1. CCL2 is an important member of the CC chemokine family which plays a crucial role in multiple CNS diseases, including stroke [7], epilepsy [8], Alzheimer's disease (AD) [9], cognitive impairment [10, 11], and HAND. Indeed, elevated CCL2 in serum and cerebrospinal fluid (CSF) has been detected in HIV-infected individuals with neurocognitive deficits [5, 12] and a high level of CCL2 in CSF was associated with higher neurocognitive deficit scores [13], suggesting that CCL2 is involved in the progression of HAND. Our previous studies showed that CCL2 enhanced NMDAR-mediated excitatory postsynaptic currents (EPSCs) in the CA1 region of rat hippocampal brain slices, injured hippocampal dendrite structure in rat hippocampal slices, and induced hippocampal neuronal death in primary culture [14]. All these previous data revealed that CCL2 may provoke excitotoxicity in its excessive excitement through NMDAR [14] and suggest a potential role of CCL2 in HIV-1-associated neuropathology. Numerous studies strongly suggest that increased CCL2 in the CNS is responsible for HIV-related encephalitis (HIVE). CCL2 is overproduced during the HIVE and accumulates in the CSF and brains of immunocompromised patients with HIV-related dementia [13]. As mentioned above, CCL2 is closely related to HAND. With this knowledge, we injected CCL2 into the bilateral hippocampi of rats to establish a cognitive disorder model as a potential mechanism for HAND. The Morris water maze (MWM) and novel object recognition tests (NORT) were used to assess the learning memory and cognition scores of the rats, respectively. Although the exact mechanism of HAND is unknown, many studies have shown that the high incidence of cognitive impairment is due to oxidative stress and inflammation [15], each of which plays an important role in the development of cognitive impairment. It has been reported that oxidative stress induces oxidative damage through apoptotic and inflammatory factors leading to cell death and to learning, memory, and cognitive impairments [16]. Indeed, our recent study showed that intrahippocampal injection of CCL2 impaired learning memory and cognition in rats via apoptosis of hippocampal neurons [17], and rats with CCL2-induced cognitive disorder could be used as an ideal murine model to study the effect of CCL2 on HAND.

Thus, drugs that inhibit or block CCL2-induced cognitive impairment could treat the cognitive dysfunction of HAND patients or improve their learning and memory, which show good prospects for clinical application. However, neuropharmacological research into drugs affecting CCL2 is still very limited. Tanshinone IIA (Tan IIA) is an important active lipophilic component extracted from the root of *Salvia miltiorrhiza Bunge* (or danshen) and exerts multiple neuroprotective effects [18, 19]. It has been reported that Tan IIA improves behavioral alterations and cognitive impairments in A*β*_1–42_-induced AD models [20]. Tan IIA also ameliorates learning and memory impairments induced by diazepam and MK-801 through the activation of ERK signaling [21]. A recent study has reported that Tan IIA has a potentially neuroprotective effect against neuronal damage via exerting significantly antioxidative activity and against a prooxidative challenge, thereby ameliorating hypoxic-ischemia-induced motor and cognitive impairments in neonatal rats [22]. However, the involvement of the CCL2-related signaling pathway in Tan IIA-induced protection of learning, memory, and cognition impairment remains unclear, and details of the underlying mechanisms remain unknown. In this study, a rat model of CCL2-induced cognitive dysfunction successfully established by injecting CCL2 into the bilateral hippocampus of rats in our previous study was employed to determine the effects of Tan IIA on CCL2-induced cognitive dysfunction, which shows a potential therapeutic approach of Tan IIA for HIV-associated neurocognitive disorder.

## 2. Materials and Methods

### 2.1. Animals and Reagents

This study was performed in accordance with the guidelines set by the Ethical Committee of Guangxi Medical University. Healthy Sprague-Dawley (SD) rats (weight: 200 ± 20g) were purchased from the Experimental Animal Center of Guangxi Medical University (Nanning, China; experimental animal production license: SCXK Gui 2014-0002, experimental animal use license: SCXK Gui 2014-0003). Rats were housed at a constant temperature (25 ± 2°C) and under a regular light-dark cycle (light on at 9:00 AM and off at 5:00 PM) with free access to water and food. The following chemicals were used: Tanshinone IIA (Nanjing Jingzhu Biotechnology; Nanjing, China); CCL2 (R&D Systems; Minneapolis, MN; catalog number: 279-MC/CF); memantine (Sigma-Aldrich; St. Louis, MO); caspase-3, caspase-8, caspase-9, IL-1*β*, IL-6, and GAPDH primers (Shanghai Jierui Bioengineering, Shanghai, China); AxyPrep Total RNA preparation kit (Corning Life Sciences, Tewksbury, MA; batch number: 07418KD1); PrimeScript RT Reagent Kit with gDNA Eraser (Takara, Kusatsu, Japan; catalog number: RR047A); TB Green Premix Ex Taq II (Tli RNase H Plus) (Takara, Kusatsu, Japan; catalog number: RR820A); kits for detection of malondialdehyde (MDA), superoxide dismutase (SOD), and glutathione peroxidase (GSH-PX) (Nanjing Jiancheng Bioengineering Institute, Nanjing, China); and a bicinchoninic acid (BCA) protein concentration assay kit (Biyuntian, Shanghai, China; catalog number: P0010).

### 2.2. Instruments

The instruments used were as follows: brain stereotaxic instrument (Shenzhen Rui wode Life Technology, Shenzhen, China), 10 *μ*L Hamilton microinjector (Hamilton, Shanghai, China; model: 701N), and Morris water maze experimental device and analysis system (Huaibei Zhenghua Biological Instrument Equipment, Huaibei, China).

### 2.3. Group and Model Preparation

**Figure 1:**
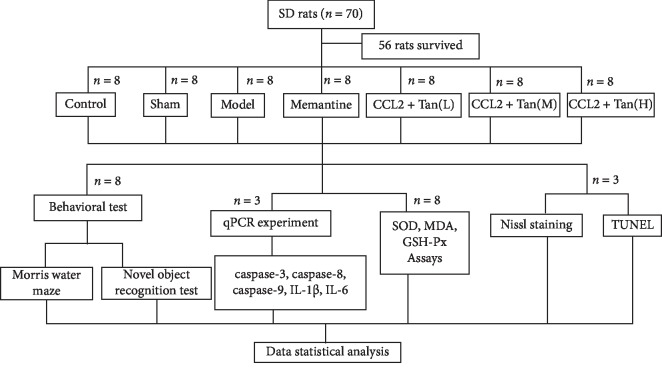
Experimental design.

Seventy male rats were randomized into seven groups: a control group (Ctrl), a sham surgery group (saline), a model group (5 ng CCL2), a positive control group (5 ng CCL2 + 10 mg/kg/d memantine), and Tanshinone IIA treatment groups: low dose (L): 5 ng CCL2 + 25 mg/kg/d Tanshinone IIA, middle dose (M): 5 ng CCL2 + 50 mg/kg/d Tanshinone IIA, and high dose (H): 5 ng CCL2 + 75 mg/kg/d Tanshinone IIA, with ten rats in each group. Tan IIA was prophylactically administrated once a day for 3 days before surgery. CCL2 was first formulated into a mother liquor of 100 mg/L and diluted with sterile physiological saline to 1 mg/L. All rats apart from those in the control group underwent stereotactic injection to the bilateral hippocampus. The injection volume per side was 2.5 *μ*l, and the sham surgery group was given an equal amount of sterile physiological saline. The rats were placed in a brain stereotaxic instrument after they were anesthetized with 10% chloral hydrate (300 mg/kg). The fur on the head of the rat was removed, and a 2 cm incision was made in the middle of the top of the head to expose the anterior fontanel. For bilateral hippocampus infusion, anterior/posterior coordinates (−3.7 mm), medial/lateral coordinates (±3.0 mm), and dorsal/ventral coordinates (−3.0 mm) (from bregma and dura, flat skull) were selected according to the stereotaxic atlas of Franklin and Paxinos. A sterilized needle connected to a Hamilton syringe was used to inject 2.5 *μ*l CCL2 into the bilateral hippocampus by intrahippocampal injection at a rate of 0.3 *μ*l/min. An equal volume of sterile physiological saline was infused as a vehicle control. The needle was retained for 5 minutes after CCL2 or sterile physiological saline injection, then the needle was slowly removed, and the pinhole was covered with a medical gelatin sponge to prevent the injection from overflowing. Penicillin was delivered, the incision was disinfected, the skin was stitched with surgical sutures, and the skin was disinfected again after aligning the incision with the skin using surgical tweezers. All rats were then treated according to the experimental design (Figure 1).

### 2.4. Behavioral Tests

#### 2.4.1. Morris Water Maze

Spatial learning and memory was tested using the Morris water maze (MWM) [23], performed 3 days after surgery. The apparatus consisted of a circular pool 160 cm across, standing 60 cm tall, and 42 cm deep. The water temperature was maintained at 22∼24°C, and the pool was divided into four equal quadrants, with a different shaped marker placed at the center of each quadrant pool. The escape platform (15 cm across, 2.0 cm below the water surface) was placed in the center of the third quadrant. The environment was kept quiet during the test. Rats were allowed to adapt to the water maze for 60 s from any quadrant in a pool without a platform 24 hours before the first spatial learning trial. For each daily trial, there were four sequential training trials beginning with placing the animal in the water facing the wall of the pool, changing the drop location for each trial. The swimming pathways, swimming speed, and latencies were recorded each day. During each trial, rats that failed to reach the hidden platform within 90 s were guided to the platform where they remained for 30 s; their latency was recorded as 90 s. The rats were subjected to 4 trials per day for 5 consecutive days, placing the rats in a different quadrant each time. On day 6, the rats were allowed to swim freely in the pool for 60 s without the platform. The crossing times of each group of rats were recorded [24].

#### 2.4.2. Novel Object Recognition Test

A novel object recognition test [25, 26] (NORT) was performed after the end of the MWM experiment. The experimental procedure consisted of three sessions: habituation, training, and retention. On the first day, each rat was habituated in a box (60 × 40 × 80 cm), allowing for 5 min of exploration in the absence of objects in order to adapt to the environment (habituation). The next day, during the training session, two jars of the same color and shape were set as familiar objects and placed at the back corner of the box. A rat was then placed in the box and the total time spent exploring the two objects was recorded for 10 min. After a training session interval of 1 h, rats entered the retention session. During the retention session, rats were placed back in the same box, in which one of the familiar objects used during the training was replaced with a novel object (a conical flask with a different color and shape from the familiar object). The animals were then allowed to explore freely for 5 min and the exploration time for the familiar (TF) and the new object (TN) during the test phase was recorded. Exploration was defined as when the rat's nose was less than 2 cm away from the object or when the rat directly touched an object with its nose. Memory was defined by the recognition index (RI) for the novel object using the following formula: RI = TN/(TN + TF).

### 2.5. qPCR

After the NORT, hippocampi of each group were dissected at day 10, and total RNA was extracted according to the instructions in the RNA extraction kit. The RNA was reverse transcribed into cDNA according to the instructions in the reverse transcription kit. Amplification of target genes was carried out to obtain the amplification curve and melting curve of target genes, and the Ct value of each gene was recorded. GAPDH was used as an internal reference gene, and the primer sequences are shown in Table 1. The results were calculated using the 2^−ΔΔCt^ method.

### 2.6. MDA, SOD, and GSH-Px Assays

The rats in each group were rapidly decapitated, and hippocampal tissues were dissected on ice. A 10% hippocampal homogenate was prepared by adding 1 : 9 physiological saline and centrifuged at 12,000×*g* for 10 min at 4°C. The supernatant was used to detect MDA levels, SOD activity, and GSH-Px activity according to the instructions of the relevant kit.

### 2.7. Nissl Staining

Rat brains were fixed for 1 h by transcardial perfusion with 4% paraformaldehyde. After deparaffinization and rehydration, the brain slices were stained with methyl violet solution for 10∼20 min followed by rinsing in distilled water. Nissl differentiation was used to differentiate for 4∼8 s until most of the staining was eliminated. Then, the slices were transferred directly from absolute ethanol to xylene. Finally, the sections were observed under a microscope and images were collected for analysis.

### 2.8. Terminal Deoxynucleotidyl Transferase-Mediated dUTP End Labeling (TUNEL) Staining

TUNEL staining was performed using the Colorimetric TUNEL Apoptosis Assay Kit (Biyuntian, Shanghai, China; catalog number: C1098). The serial 7 *μ*m thick coronal paraffin sections were dewaxed with xylene, treated with proteinase K, and rinsed with PBS. Endogenous peroxidase activity was blocked by incubating with 0.3% H_2_O_2_ in PBS for 10 min at room temperature. The sections were then incubated in 50 *μ*l TUNEL solution for 1 h at 37°C. Then, the sections were incubated in a streptavidin-HRP solution and stained with DAB. After counterstaining with hematoxylin, the sections were examined with an optical microscope. TUNEL-positive CA1 neurons were carefully counted in three sections per animal. Cell counts from each of the three hippocampal sections were averaged to provide a mean value.

### 2.9. Statistical Analysis

Statistical analyses of the data were performed in SPSS 17.0 (IBM, Armonk, NY), and all results are expressed as mean ± standard error of the mean (SEM). For the MWM tests, escape latency, swimming pathways, and swimming speed in the hidden platform trial were analyzed by two-way ANOVA of repeated measures, followed by Tukey's post hoc test for multiple comparisons. The other data were analyzed by one-way ANOVA. Significance was defined as *p* < 0.05.

## 3. Results

### 3.1. Effects of Tan IIA on Spatial Learning and Memory Impairment Induced by CCL2

**Figure 2:**
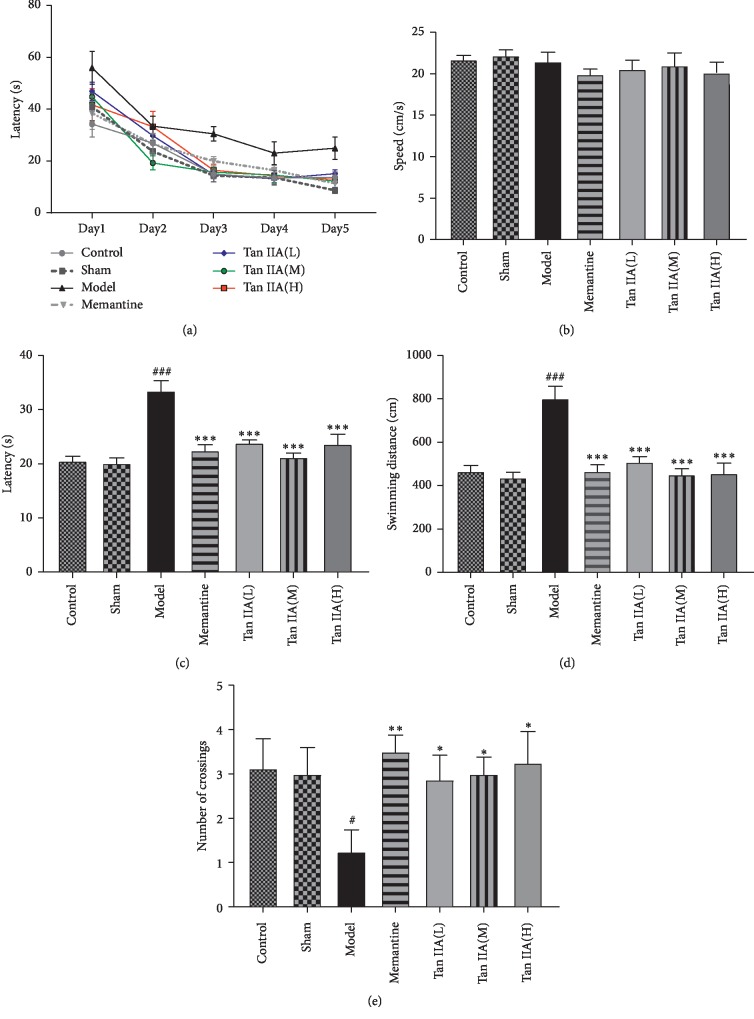
Effect of Tanshinone IIA on learning and memory deficits in CCL2-induced rats. (a) Escape latency tendency of five consecutive days' test. (b) The swimming speed among the groups. As shown, the speed was not different among the groups. (c) Escape latency of finding the hidden platform in the probe trial. Tan IIA treatment groups (Tan IIA(L): 25 mg/kg, Tan IIA(M): 50 mg/kg; Tan IIA(H): 75 mg/kg) spent less time to find hidden platform compared with the model group. (d) Swimming distance of finding the hidden platform in the probe trial. The Tan IIA treatment groups showed a significantly shorter distance to arrive platform. (e) The number of platform crossings in the probe test. All data are expressed as the mean ± SEM (*n* = 8, ^*∗*^*p* < 0.05, ^*∗∗*^*p* < 0.01, and ^*∗∗∗*^*p* < 0.001 versus model; ^#^*p* < 0.05, ^###^*p* < 0.001 versus sham).

**Figure 3:**
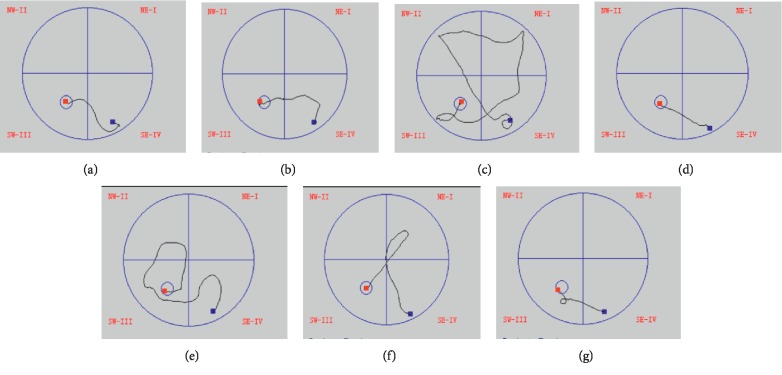
Typical swim-tracking path in probe trial on the fifth training day. Tan IIA treatment groups showed a swim-tracking path shorter than model group. (a) Control. (b) Sham. (c) Model. (d) Memantine. (e) Tan IIA(L). (f) Tan IIA(M). (g) Tan IIA(H).

To establish a spatial learning and memory impairment model, we infused CCL2 (5 ng) into the bilateral hippocampus of rats and measured the spatial learning and memory deficits by the MWM test. To study the effects of Tan IIA on CCL2-induced spatial learning and memory deficits, the cognitively impaired rats were treated with different doses of Tan IIA by intragastric administration (25, 50, and 75 mg/kg; represented by Tan IIA(L), Tan IIA(M), and Tan IIA(H), respectively). As shown in Figure 2(b), there was no significant difference in swimming speed among the groups (*F*_(6 49)_ = 0.634, *p* > 0.05). In Figure 2(a), the time for rats to find the hidden platform declined progressively over the course of the five training days. In Figures 2(c) and 2(d), there was no difference in escape latency or swimming distance between the control and sham groups, indicating that brain stereotactic injection alone has no effect on rats. Compared to the sham group, escape latency (*F*_(2 21)_ = 35.42, *p* < 0.001) and swimming distance (*F*_(2 21)_ = 28.80, *p* < 0.001) increased in the model group. However, pretreatment with different doses of Tan IIA clearly shortened escape latency (*F*_(4 35)_ = 14.12, *p* < 0.001) and swimming distance (*F*_(4 35)_ = 15.59, *p* < 0.001) in contrast to the model group. In Figure 2(e), on the sixth day, the probe trial was performed by removing the platform and allowing rats to swim freely to estimate their spatial-working memory. The model group crossed the position of the removed platform fewer times than controls, while this phenomenon was ameliorated by pretreatment with Tan IIA. The corresponding swimming paths of each group on the fifth trial day are shown in Figure 3. The model group presented a more chaotic and longer swimming path than controls, which were improved by Tan IIA pretreatment. Taken together, these results indicate that Tan IIA pretreatment is able to prevent CCL2-induced deficits of spatial learning and memory.

### 3.2. Effects of Tan IIA on Cognitive Function Impairment Induced by CCL2

**Figure 4:**
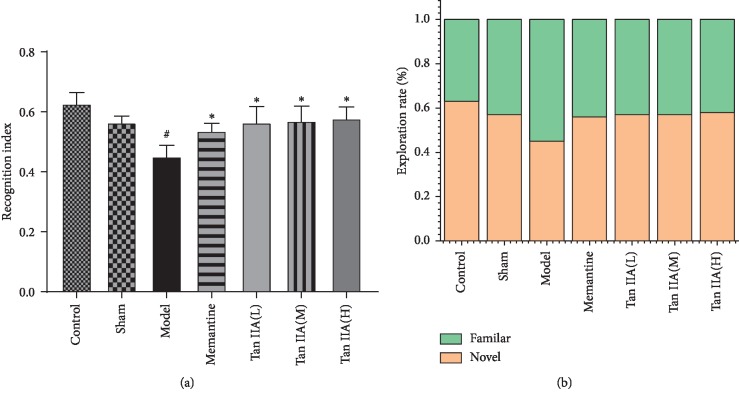
Effect of Tanshinone IIA on the recognition memory in CCL2-induced cognition deficits rats detected by a novel object recognition test. (a) Discrimination index of each group of rats. As is shown, Tan IIA groups (Tan IIA(L): 25 mg/kg, Tan IIA(M): 50 mg/kg; Tan IIA(H): 75 mg/kg) obviously increased the discrimination index of cognition deficits rats. (b) The percentage of the time explored familiar and novel object. Tan IIA treatment groups spent more time to explore the novel object when compared to the model group. The data are expressed as mean ± SEM. The analysis was performed using one-way ANOVA (*n* = 8, ^*∗*^*p* < 0.05 versus model, ^#^*p* < 0.05 versus sham).

In order to evaluate cognitive function, a novel object recognition test was carried out in each group of rats. As shown in Figure 4(a), compared with the sham group, the recognition index was significantly reduced in model rats (*p* < 0.05). Tan IIA clearly increased the recognition index of cognitively impaired rats. There was a significant difference in the recognition index between Tan IIA groups and the model group (*p* < 0.05). As shown in Figure 4(b), Tan IIA treatment groups spent more time exploring the novel object compared to the model group.

### 3.3. Effects of Tan IIA on Oxidative Stress Status in Learning and Memory Deficits Rats Induced by CCL2

**Figure 5:**
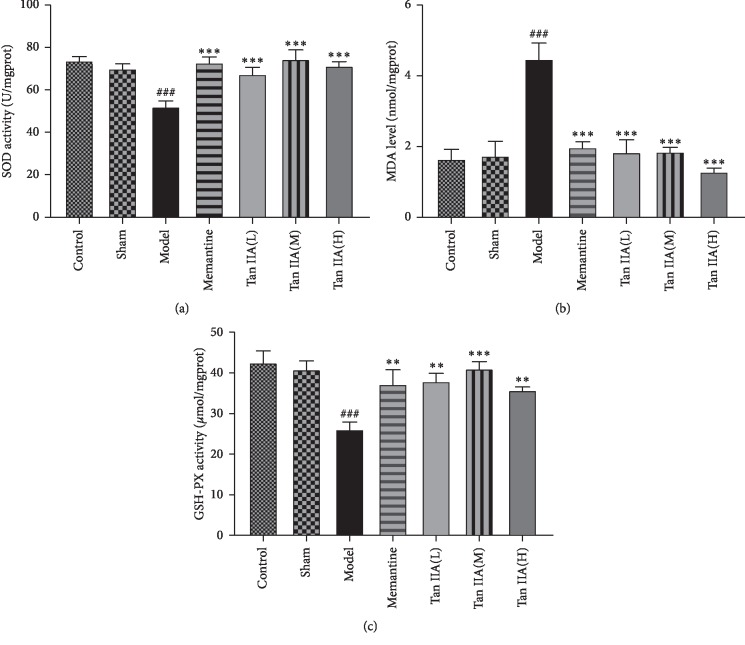
Effect of different doses of Tan IIA 25, Tan IIA 50, and Tan IIA 75 (mg/kg) on the oxidative stress status in CCL2-treated rats. The supernatant of the hippocampus homogenate was used for the assay of SOD, GSH-PX activity, and MDA levels. A significant increase in (a) SOD and (c) GSH-PX activity in Tan IIA treatment groups (Tan IIA(L): 25 mg/kg, Tan IIA(M): 50 mg/kg; Tan IIA(H): 75 mg/kg) compared with model group. (b) A significant reduction in MDA levels in Tan IIA treatment groups compared with the model group. All data are expressed as the mean ± SEM (*n* = 8, ^*∗∗*^*p* < 0.01, ^*∗∗∗*^*p* < 0.001 versus model, ^###^*p* < 0.001 versus sham).

As shown in Figures 5(a)–5(c), there were no significant differences in MDA levels or SOD and GSH-Px activity between the sham and control groups. Compared with the sham group, SOD and GSH-Px activity was robustly decreased in the model group (*p* < 0.001), while the MDA level was increased (*p* < 0.001). The Tan IIA treatment groups had increased SOD and GSH-Px activity and decreased MDA levels compared to the model group (*p* < 0.001 and *p* < 0.001, respectively).

### 3.4. Effects of Tan IIA on IL-1*β* and IL-6 mRNA Expression in CCL2-Treated Rat Hippocampus

**Figure 6:**
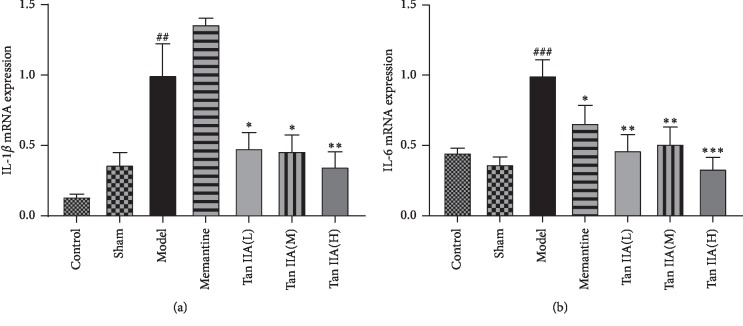
Effects of Tanshinone IIA on IL-1*β* and IL-6 mRNA expression in the hippocampus of CCL2-treated rats. Quantitative real-time PCR analysis of messenger (m) RNA levels. Relative expression of (a) IL-1*β*mRNA and (b) IL-6mRNA. qPCR analysis showed increased expression of IL-1*β* and IL-6 mRNA in the hippocampus of model rats. This result showing that Tanshinone IIA (Tan IIA(L): 25 mg/kg, Tan IIA(M): 50 mg/kg; Tan IIA(H): 75 mg/kg) significantly decreased the expression of IL-1*β* and IL-6 mRNA in model group rat. The experiments were repeated three times independently. All data are expressed as mean ± SEM (*n* = 3, ^*∗*^*p* < 0.05, ^*∗∗*^*p* < 0.01, ^*∗∗∗*^*p* < 0.001 versus mode; ^##^*p* < 0.01, ^###^*p* < 0.001 versus sham).

As shown in Figures 6(a)–6(b), brain stereotactic injection of CCL2 remarkably increased the expression of IL-1*β* and IL-6 mRNA in the hippocampus. Compared with the model group, Tan IIA significantly downregulated IL-1*β* and IL-6 mRNA expression (*p* < 0.05 and *p* < 0.01, respectively). In addition, there was an interaction between CCL2 and Tan IIA in terms of IL-1*β* and IL-6 mRNA expression, suggesting that Tan IIA may inhibit CCL2-induced upregulation of IL-1*β* and IL-6 mRNA.

### 3.5. Tan IIA Rescues Neuronal Damage in CA1 Region of the Hippocampus in Rats Treated with CCL2

**Figure 7:**
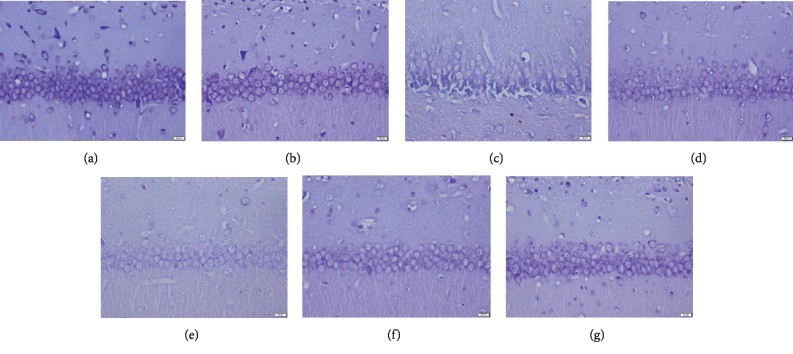
Effect of Tan IIA on the hippocampal neuron damage in the hippocampus induced by CCL2. The CCL2 induces neuron damage and cell loss in the hippocampal CA1 regions, and the phenomenon can be rescued by Tan IIA. The hippocampus Nissl staining analysis was performed after the end of the novel object recognition test (Tan IIA(L):25 mg/kg, Tan IIA(M): 50 mg/kg; Tan IIA(H): 75 mg/kg). (a) Control. (b) Sham. (c) Model. (d) Memantine. (e) Tan IIA(L). (f) Tan IIA(M). (g) Tan IIA(H).

As shown in Figure 7, CCL2 induces cell loss in the hippocampal CA1 region, while Tan IIA rescued this loss. The results indicated clear cell loss and damage in the CA1 region of the hippocampus of the model group compared to other groups. Furthermore, there were significantly more cells in the hippocampus in the Tan IIA treatment group compared to the model group.

### 3.6. Antiapoptotic Effect of Tan IIA on Hippocampal Neuron Damage Induced by CCL2

**Figure 8:**
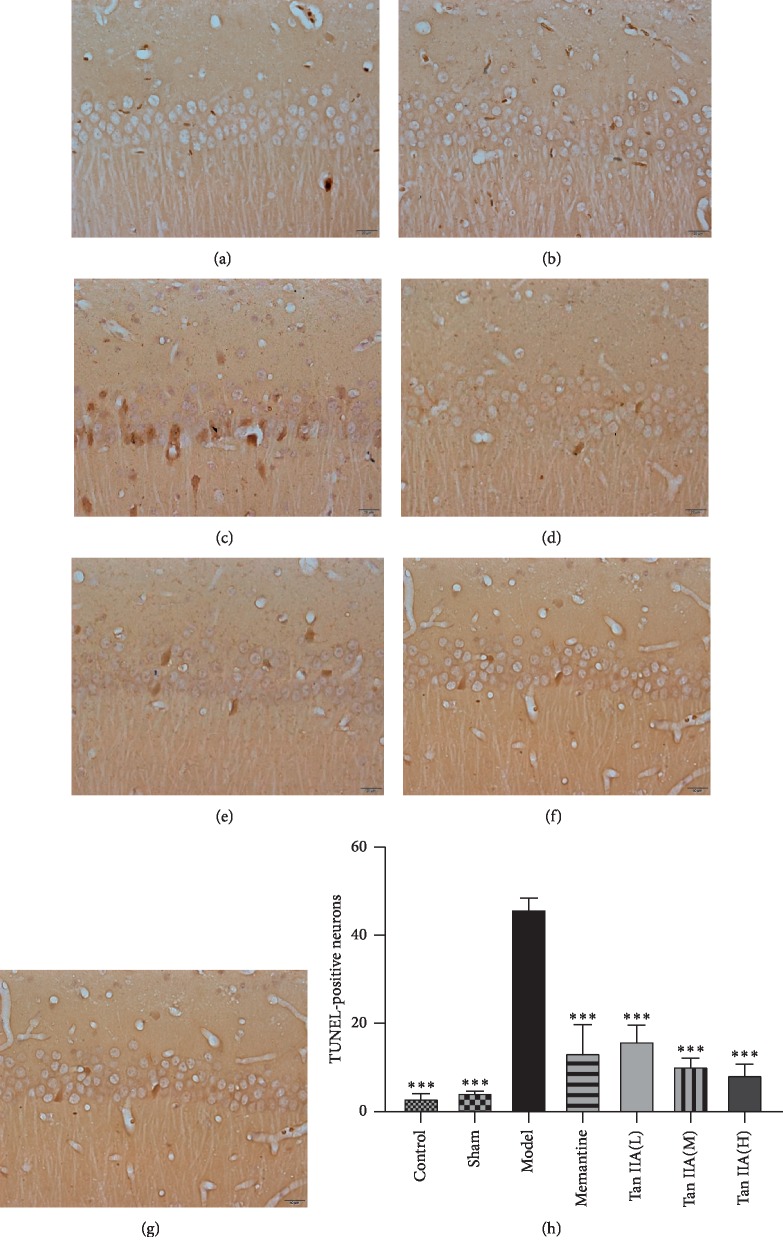
Cell apoptosis of hippocampal tissues in rats among the seven groups. (a–g) Representative 400× images of the TUNEL staining. TUNEL staining showed that Tan IIA reduced CCL2-induced cell apoptosis in hippocampus CA1 areas, and the brown nuclei represented TUNEL-positive cells. (h) Cell apoptosis rate among seven groups. The results are expressed as mean ± SEM, *n* = 3. ^*∗∗∗*^*p* < 0.001 versus model group (Tan IIA(L): 25 mg/kg, Tan IIA(M): 50 mg/kg; Tan IIA(H): 75 mg/kg). (a) Control. (b) Sham. (c) Model. (d) Memantine. (e) Tan IIA(L). (f ) Tan IIA(M). (g) Tan IIA(H).

**Figure 9:**
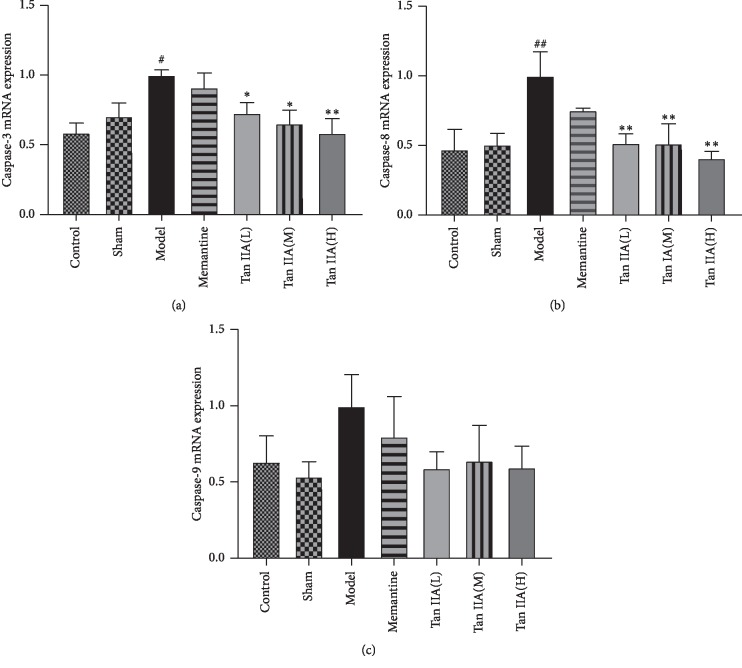
Effects of Tanshinone IIA on caspase-3, caspase-8, and caspase-9 mRNA expression in the hippocampus of CCL2 treated rats. Quantitative real-time PCR analysis of messenger (m) RNA levels. Relative expression of (a) caspase-3 mRNA, (b) caspase-8 mRNA, and (c) caspase-9 mRNA. qPCR analysis showed increased expression of caspase-3, caspase-8, and caspase-9 mRNA in the hippocampus of model rats. Caspase-3, caspase-8, and caspase-9 mRNA expression in the Tanshinone IIA treatment groups (Tan IIA(L): 25 mg/kg, Tan IIA(M): 50 mg/kg; Tan IIA(H): 75 mg/kg) decreased compared to the CCL2 group (*p* < 0.05, *p* < 0.01) but showed no statistically significant difference between the model and Tanshinone IIA treatment groups on caspase-9 mRNA expression (*p* > 0.05). The experiments were repeated three times independently. All data are expressed as mean ± SEM (*n* = 3, ^*∗*^*p* < 0.05, ^*∗∗*^*p* < 0.01 versus model; ^#^*p* < 0.05, ^##^*p* < 0.01 versus sham).

Using TUNEL staining, the number of apoptotic CA1 hippocampal cells was shown to have increased in the model group, while the Tan IIA group had fewer apoptotic CA1 hippocampal cells (Figures 8(a)–8(g)). When comparing the numbers of TUNEL-positive neurons among different groups (Figure 8(h)), there were significantly fewer TUNEL-positive neurons in the Tan IIA group compared to the model group (*p* < 0.001). We also tested the mRNA levels of the apoptosis-related genes caspase-3, caspase-8, and caspase-9. qPCR analysis showed that the expression of caspase-3, caspase-8, and caspase-9 varied significantly among groups. As shown in Figures 9(a)–9(c), the expression of caspase-3, caspase-8, and caspase-9 in the model group was higher than that of other groups. Compared to the model group, the expression of caspase-3, caspase-8, and caspase-9 decreased in the Tan IIA treatment group (*p* < 0.05 and *p* < 0.01, respectively). However, there was no difference in caspase-9 expression between the Tan IIA treatment group and model group (*p* > 0.05). These results demonstrated that Tan IIA could protect against CCL2-induced learning and memory deficits through an antiapoptosis mechanism.

## 4. Discussion

With the global outbreak of AIDS, HAND has gradually been recognized and studied, and HAND is becoming another major cause of dementia following Alzheimer's disease and vascular dementia. Meanwhile, with the growth of the aging population, the incidence of cognitive disorders is also increasing. Mild cognitive deficits are an effective predictor of dementia [27]. Therefore, HIV-1-induced cognitive disorders are getting more and more attention. HIV-1 not only attacks the peripheral immune system but also invades the CNS to induce HIV-related neurocognitive dysfunction. Studies have shown that HIV-1 infection and immune-activated macrophages (HIV/MDM) secrete a range of neurotoxic substances, and CCL2 is an important component of HIV/MDM secretion [28, 29]. CCL2 is a member of the CC chemokine family and is also known as monocyte chemoattractant protein 1 (MCP-1). CCL2 is one of the most studied CC chemokines [30]. It plays an important role in the CNS where it can induce and participate in various neurodegenerative diseases. The biological role of CCL2 is mainly produced by its G-protein coupled receptor CCR2 [31, 32]. Studies have also shown that CCL2 expression is significantly higher in the cerebrospinal fluid of HIV-1-infected individuals than that in healthy subjects, and the number of receptors with a high affinity for CCL2 is also significantly upregulated after HIV-1 infection [33]. In addition, some reports have shown a possible association between CCL2 levels in CSF and the development of HIV-associated dementia, suggesting that this chemokine could be used as a biomarker of disease progression [34]. Therefore, CCL2 is extremely critical in promoting the development of HAND/HAD. Meanwhile, it has been reported that CCL2 is not only overexpressed in the CSF of patients with HAND [10] but is also overexpressed in the CSF of patients with cognitive impairment in Parkinson's disease [35]. As such, we suspect that cognitive impairment is associated with CCL2 and that CCL2 may be an important pathogenic factor of HAND and other cognitive impairment diseases. Currently, however, research on therapeutic drugs for neurocognitive impairment targeting CCL2 is very limited. Thus, our study intended to find a potential drug against CCL2 based on previous research on the mechanism of CCL2 in central pathogenesis.

Tan IIA is a liposoluble diterpene ketone compound extracted from a plant used in traditional Chinese medicine, *Salvia miltiorrhiza*. It has been widely used in the prevention and treatment of cardiovascular diseases such as coronary heart disease and angina pectoris. In addition, recent studies have found that Tan IIA has neuroprotective effects, and so we designed an experiment to investigate whether Tan IIA can protect CCL2-induced cognitive dysfunction in the nervous system. In addition, our previous studies have found that CCL2 could impair the structure of hippocampal CA1 neuron dendrite in hippocampal slice and induce primary culture of hippocampal neurons death via NMDAR [14]. Hence, we also treated rats with memantine, a noncompetitive NMDAR antagonist, to further confirm the role of NMDAR in CCL2 pathological effects.

In our study, in order to explore the neuroprotective mechanisms of Tan IIA in CCL2-induced cognitive dysfunction treatment, we established a rat model of cognitive dysfunction by infusing CCL2 into the bilateral hippocampus of rats. In the behavioral experiment, our study revealed that the model group had a longer latency and swimming distance in an MWM and crossed the area of the removed platform fewer times than controls, and a lower recognition index, which demonstrated that the learning and memory ability of the model group of rats was significantly reduced and that CCL2 can induce cognitive dysfunction. Furthermore, Tan IIA reversed the phenomena of learning and memory deficits when the rats were treated with different doses of Tan IIA. These behavioral test results suggest that Tan IIA has a significant protective effect on CCL2-induced learning memory and cognitive impairment in rats. In addition, the results were similar in the memantine group, which confirmed the involvement of NMDAR in learning and memory deficits by CCL2.

Oxidative stress refers to the production of reactive oxygen species (ROS) and reactive nitrogen species (RNS) by the body due to harmful stimuli from the internal and external environment, which cause physiological and pathological responses. Under physiological conditions, a variety of antioxidant enzymes can promptly remove ROS and RNS that are overproduced and released due to various reasons to maintain the body's redox balance process. When the function of the antioxidant enzyme system is reduced or the body's oxidation products are excessively increased, resulting in an imbalance of redox balance leading to cell and tissue damage, oxidative stress damage will be induced. Lee found that using CCR2 knockout mice to block CCL2-CCR2 signaling could ameliorate obesity-induced albuminuria in the kidney through blocking oxidative stress. In another study, researchers revealed that CCL2 deficiency could decrease hepatic liver oxidative stress, which suggested that CCL2 may involve in oxidative stress [36]. Our present study showed that CCL2 significantly decreased SOD and GSH-Px activity and increased MDA in the hippocampus. Significant impairments in learning and memory were observed in CCL2-treated rats in comparison to controls. Hence, it can be inferred that sustained oxidative stress is one of the possible mechanisms by which CCL2 causes cognitive dysfunction in rats. Oxidative stress has been closely associated with neurodegenerative disorders, where neuronal damage is considered to be primarily related to free radical scavenging ability in the body. A decrease in antioxidant capacity and excessive ROS can cause lipid peroxide metabolite MDA accumulation [37], damaging the cell membrane of hippocampal neurons and leading to learning and memory dysfunction. Thus, learning memory and cognitive impairment can be indirectly assessed by changes in activities such as SOD, MDA, and GSH-Px. SOD and GSH-Px are important antioxidant enzymes that are ubiquitous in the body. SOD can effectively remove superoxide anion radicals (O^2−^) produced by biological oxidation [38]. GSH-Px catalyzes the conversion of GSH to glutathione disulfide (GSSG), which reduces toxic peroxides to nontoxic hydroxy compounds and promotes the decomposition of H_2_O_2_ [39]. They can protect membrane structure and cell function by terminating the free radical chain reaction. Our results showed that Tan IIA conspicuously increased SOD and GSH-Px activity and reduced MDA levels in the hippocampus. These results indicated that Tan IIA significantly enhanced the level of antioxidative activity in the CCL2-treated rat hippocampus.

Researches have demonstrated that [40, 41] the application of memantine could reduce histone deacetylase activity and increase glial-derived neurotrophic factors, which may lead to decrease oxidative stress and nitrosative stress in the brain [42]. In addition, it has been shown that intracellular calcium overload through NMDAR would impair mitochondrial function, which leads to the formation of ROS. Thus, memantine, as a noncompetitive NMDAR antagonist, has an ability against oxidative stress, such as reducing MDA and increasing SOD and GSH-Px via inhibiting the influx of calcium.

Inflammation and oxidative stress usually occur simultaneously. Increased levels of oxidative stress can promote the production of TNF-*α*, IL-1*β*, and other inflammatory cytokines. These inflammatory cytokines can stimulate free radical production, thereby increasing the level of oxidative stress, leading to neuronal death [43]. Chemokines are some of the most important inflammatory factors. CCL2 is one of the most studied proinflammatory molecules among CC chemokines [30]. It can induce the expression of various inflammatory cytokines and chemokines, amplify the inflammatory response, and damage tissue cells. When microglia are destroyed and activated by inflammatory stimuli, they secrete a large number of proinflammatory cytokines, such as IL-1*β* and IL-6, to promote the production of inducible NO synthase (iNOS) by nerve cells and cause neuronal death [44, 45], leading to cognitive dysfunction. For example, in a mouse model of kainic acid- (KA-) induced epilepsy, it was found that CCL2 expression in the hippocampus increased significantly after 24 hours of lateral ventricular injection of KA. Degeneration and death of hippocampal CA3 neurons were also observed with the activation of microglia. Further studies suggest that CCL2 may induce neuronal damage by activating STAT3 to induce IL-1*β* production [8]. Evidence shows that neuroinflammation can induce and aggravate neurodegenerative diseases [46]. In addition, studies have long shown that inflammatory processes in the brain are associated with impaired cognitive function. Tanshinone IIA, an active compound, has multiple pharmacological functions, such as anti-inflammation and antioxidation. After the exposure of Tanshinone IIA to atherosclerosis model which is established by THP-1-derived macrophages treated with ox-LDL, the expression of IL-1*β*, IL6, and TNF-*α* was decreased compared to the untreated group, and a further study revealed that Tanshinone IIA may decrease the inflammatory response by mediating miR-130b and WNT5A [47]. In this study, our results showed that CCL2 significantly upregulated the expression of IL-1*β* and IL-6 in hippocampi of rats, while the expression of IL-1*β* and IL-6 in hippocampi of rats pretreated with Tan IIA decreased. Thus, pretreatment with Tan IIA significantly prevented cognitive impairment induced by CCL2 and inhibited the expression of IL-1*β* and IL-6. These results suggest that Tan IIA has clear anti-inflammatory properties in rats with CCL2-induced cognitive dysfunction.

Memantine shows anti-inflammation properties via reduction of calcium influx, B-cell receptor signaling, toll-like receptor 4 signaling, and activation of Erk1/2, Akt, and NFATc1 pathways [48]. Indeed, a few studies have found that treatment with memantine caused a reversal of the increase of IL-1*β* and TNF-*α* in the hippocampus and cortex induced by streptozotocin [49]. In addition, memantine treatment showed lower IL-6 expression in the hippocampus of olfactory bulbectomized mice, and the underlying mechanism may link to PKA-ERK-CREB-BDNF/Bcl-2-caspase-3 pathway [50]. In our current results, we found that treatment with memantine could significantly decrease IL-6 mRNA expression. However, the mRNA expression of IL-1*β* is unexpectedly higher than the model group. We considered that the dose of memantine may play a key role in it. A research found that 20 mg/kg memantine could decrease the level of IL-1*β* in the hippocampus in a model of intracerebroventricular colchicine-injected rats [51]. Thus, it is necessary to further explore the anti-inflammation property of 10 mg/kg memantine in our experimental condition.

Oxidative stress and inflammatory responses can directly lead to apoptosis. Our results showed that Tan IIA remarkably downregulated the expression of caspase-3, caspase-8, and caspase-9 in the hippocampi of CCL2-treated rats. In addition, TUNEL staining showed that Tan IIA markedly attenuated hippocampal neuronal apoptosis in CCL2-induced rats. Neuronal apoptosis may be one of the main factors leading to cognitive dysfunction. Apoptosis, or programmed cell death, is cell death driven by a signaling cascade of proteins and is considered to be one of the main causes of neurodegenerative disease [52]. Apoptosis is divided into a caspase-dependent apoptotic pathway and a noncaspase-dependent apoptotic pathway. In addition, the caspase-dependent pathway is further divided into two: the death receptor pathway and the mitochondrial pathway. Caspase-3 is a proapoptotic enzyme that is a central link and executor of apoptosis and plays an important role in these two pathways [53]. Caspase-8 is a key promoter in the death receptor-mediated apoptotic pathway. Caspase-8 is activated by self-cleavage by oligomerization and activates downstream caspases to produce an apoptotic effect. Caspase-9 is an important initiating factor in the cell mitochondrial apoptotic pathway. In the mitochondria, the caspase-9 precursor is cleaved by cytochrome C and released into the cytoplasm, where it finally activates caspase-9 to initiate apoptosis [54]. Our results suggest that the protective effect of Tan IIA against CCL2-induced cognitive dysfunction is related to the downregulation of the proapoptotic enzymes caspase-3, caspase-8, and caspase-9.

## 5. Conclusions

**Figure 10:**
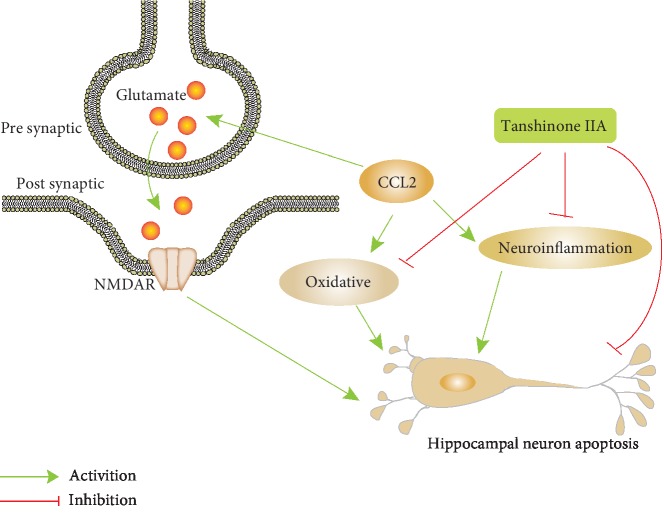
The schematic diagram of our hypothesis. Tan IIA can protect CCL2-induced learning memory and cognition impairment, and its mechanism is related to reducing brain tissue inflammation, and antioxidative stress, and finally inhibiting the excessive apoptosis of nerve cells, indicating that Tan IIA has potential nerve protective effects.

In summary, these data implicate that Tan IIA can ameliorate CCL2-induced learning memory and cognitive impairment by impacting oxidative stress and inflammation and finally inhibiting the excessive apoptosis of nerve cells (Figure 10). Thus, Tan IIA may be a potential therapeutic agent of HAND.

## Figures and Tables

**Table 1:** Primer sequences for target genes.

Gene	Primer sequence
Caspase-3	F: 5′-GCAGCAGCCTCAAATTGTTGAC-3′
	R: 5′-TGCTCCGGCTCAAACCATC-3′
Caspase-8	F: 5′-CCTGTTCTAAGCCTGTCTC-3′
	R: 5′-TGGGAAGGAAGCCTCTAT-3′
Caspase-9	F: 5′-ACGTTGTTGATGATGAGGC-3′
	R: 5′-CGGTGGACATTGGTTCTG-3′
IL-1*β*	F: 5′-AGGAGAGACAAGCAACGACA-3′
	R: 5′-CTTTTCCATCTTCTTTGGGTAT-3′
1L-6	F: 5′-AGTTGCCTTCTTGGGACTGATGT-3′
	R: 5′-GGTCTGTTGTGGGTGGTATCCTC-3′
GAPDH	F: 5′-GACATGCCGCCTGGAGAAAC-3′
	R: 5′-AGCCCAGGATGCCCTTTAGT-3′

## Data Availability

The data used to support the findings of this study are available from the corresponding author upon request.
