# Corrigendum to “Tanshinone IIA Alleviates CCL2-Induced Leaning memory and Cognition Impairment in Rats: A Potential Therapeutic Approach for HIV-Associated Neurocognitive Disorder”

**DOI:** 10.1155/2020/5834542

**Published:** 2020-12-22

**Authors:** Yuan-jun Liao, Jian-min Chen, Jiang-yi Long, Yi-jun Zhou, Bing-yu Liang, Yan Zhou

**Affiliations:** ^1^Department of Pharmacology, Guangxi Medical University, Nanning, Guangxi 530021, China; ^2^Guangxi Key Laboratory of AIDS Prevention and Treatment, Guangxi Medical University, Nanning, Guangxi 530021, China

Following the publication of the article “Tanshinone IIA Alleviates CCL2-Induced Leaning memory and Cognition Impairment in Rats: A Potential Therapeutic Approach for HIV-Associated Neurocognitive Disorder” [1], the authors identified that the incorrect images were presented in Figures 8(d) and 8(g).  Figure 8(g) was mistakenly duplicated with Figure 8(f), and Figure 8(d) was incorrectly taken from a different experiment. The authors explained that the error occurred during manuscript preparation and apologize for any inconvenience caused. The correct figure is as follows

## Figures and Tables

**Figure 1:**
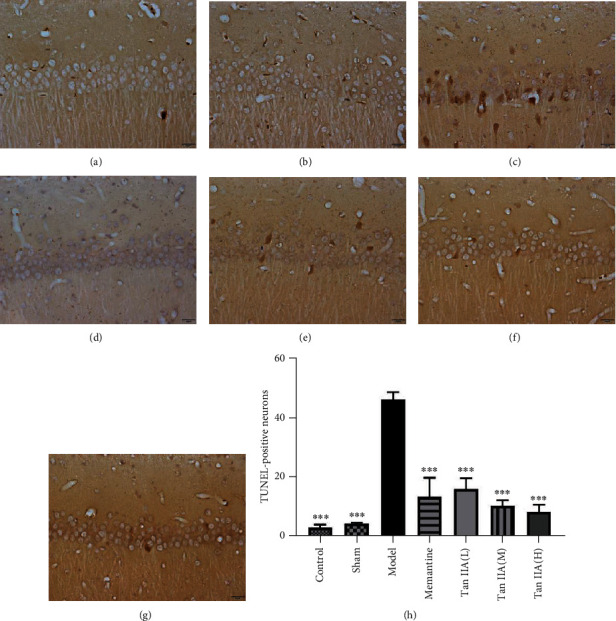
Cell apoptosis of hippocampal tissues in rats among the seven groups. (a–g) Representative 400× images of the TUNEL staining. TUNEL staining showed that Tan IIA reduced CCL2-induced cell apoptosis in the hippocampus CA1 areas, and the brown nuclei represented TUNEL-positive cells. (h) Cell apoptosis rate among seven groups. The results are expressed as mean ± SEM, *n* = 3. ^∗∗∗^*p* < 0.001 versus the model group (Tan IIA(L): 25 mg/kg, Tan IIA(M): 50 mg/kg; Tan IIA(H): 75 mg/kg). (a) Control. (b) Sham. (c) Model. (d) Memantine. (e) Tan IIA(L). (f) Tan IIA(M). (g) Tan IIA(H).
